# Mid-infrared spectroscopy combined with chemometrics to detect Sclerotinia stem rot on oilseed rape (*Brassica napus* L.) leaves

**DOI:** 10.1186/s13007-017-0190-6

**Published:** 2017-05-17

**Authors:** Chu Zhang, Xuping Feng, Jian Wang, Fei Liu, Yong He, Weijun Zhou

**Affiliations:** 1 College of Biosystems Engineering and Food Science, Zhejiang University, 866 Yuhangtang Road, Xihu District, Hangzhou, 310058 China; 2 College of Agriculture and Biotechnology, Zhejiang University, Hangzhou, 310058 China

**Keywords:** Mid-infrared spectroscopy, Second derivative spectra, Sclerotinia stem rot, Oilseed rape, Sample set validation

## Abstract

**Background:**

Detection of plant diseases in a fast and simple way is crucial for timely disease control. Conventionally, plant diseases are accurately identified by DNA, RNA or serology based methods which are time consuming, complex and expensive. Mid-infrared spectroscopy is a promising technique that simplifies the detection procedure for the disease. Mid-infrared spectroscopy was used to identify the spectral differences between healthy and infected oilseed rape leaves. Two different sample sets from two experiments were used to explore and validate the feasibility of using mid-infrared spectroscopy in detecting Sclerotinia stem rot (SSR) on oilseed rape leaves.

**Results:**

The average mid-infrared spectra showed differences between healthy and infected leaves, and the differences varied among different sample sets. Optimal wavenumbers for the 2 sample sets selected by the second derivative spectra were similar, indicating the efficacy of selecting optimal wavenumbers. Chemometric methods were further used to quantitatively detect the oilseed rape leaves infected by SSR, including the partial least squares-discriminant analysis, support vector machine and extreme learning machine. The discriminant models using the full spectra and the optimal wavenumbers of the 2 sample sets were effective for classification accuracies over 80%. The discriminant results for the 2 sample sets varied due to variations in the samples.

**Conclusion:**

The use of two sample sets proved and validated the feasibility of using mid-infrared spectroscopy and chemometric methods for detecting SSR on oilseed rape leaves. The similarities among the selected optimal wavenumbers in different sample sets made it feasible to simplify the models and build practical models. Mid-infrared spectroscopy is a reliable and promising technique for SSR control. This study helps in developing practical application of using mid-infrared spectroscopy combined with chemometrics to detect plant disease.

## Background

Oilseed rape (*Brassica napus* L.) is one of the most important sources of edible oil and biodiesel. The growth of oilseed rape, a widely planted oil-bearing crop, is affected by many factors, including seed, soil, water supply, nutritional elements, weather conditions and diseases. Diseases are major threats to oilseed rape, resulting in yield and quality loss.

Sclerotinia stem rot (SSR) is a major disease affecting the oilseed rape growth and causing severe yield loss. The ascospores of SSR are produced by the apothecia in the soil, or the seeds are discharged into the air. Some of the ascospores are dispersed more widely from other fields into the surrounding crops. The spread of ascospores makes it difficult to control the disease completely before its onset. The detection of SSRs at an early stage provides an alternative for disease control.

The early detection of SSR on oilseed rapes is a priority for SSR control on oilseed rape plants. Traditional methods, such as polymerase chain reaction (PCR) [1], enzyme-linked immunosorbent assay (ELISA) [2], nucleic acid hybridization [3] and serological techniques [4], rely on the identification of spores by microscopy or culture-based techniques to detect plant diseases. These traditional methods applied in the detection of plant diseases are accurate and standard. However, these methods also have some limitations such as being time-consuming, requiring special operation skills, generating reagent waste and requiring complex sample preparation, which makes these methods unsuitable for large-scale field detection. Thus, new techniques for cheap, fast and accurate identification of plant diseases should be developed.

Spectroscopy techniques, such as visible/near-infrared spectroscopy [5, 6], mid-infrared spectroscopy [7–9], Raman spectroscopy [10] and fluorescence spectroscopy [11] have been studied to detect plant diseases. Mid-infrared spectroscopy provides the information about the fundamental vibrational bands of the functional groups in the samples. The plants affected by diseases experience internal physiological changes, which in turn results in changes in their mid-infrared spectra. Mid-infrared spectroscopy has been used as an effective technique for detecting plant diseases. Sankaran et al. [7] used mid-infrared spectroscopy to detect nitrogen deficiency and Huanglongbing of citrus leaves. Hawkins et al. [8] used Fourier transform infrared-attenuated total reflection spectroscopy for the detection of Huanglongbing in citrus leaves. Hawkins et al. [9] also used the Fourier transform infrared-attenuated total reflection spectroscopy to detect Huanglongbing, citrus leaf rugose virus, citrus tristeza virus, citrus psorosis virus, *Xanthomonas axonopodis* and nutritional deficiency.

Moreover, the use of mid-infrared spectroscopy in plant disease detection mainly focuses on the spectral differences or the discriminant results, and the feasibility of using mid-infrared spectroscopy for plant disease detection has been proven. However, there is a wide gap between the feasibility of this technique and its practical application is great. The rapid acquisition of spectra and simple sample preparation makes it possible to develop mid-infrared spectroscopy as a practical method for the rapid detection of plant diseases. The primary purpose of developing a practical application of mid-infrared spectroscopy depends on the calibration models. Robust and accurate models using informative wavenumbers with minimum colinearity and redundancy are required.

The objective of this study was to explore and validate the use and capacity of mid-infrared spectroscopy for detecting SSRs on oilseed rape leaves. The specific objectives were: (1) to evaluate the influence of different samples sets on mid-infrared spectroscopy, (2) to select and compare optimal wavenumbers in different sample sets, and (3) to develop and compare the optimal classification models in different sample sets.

## Methods

### Sample preparation

The seeds of the oilseed rape (*Brassica napus* L., cv. ZS758) were used in our study. The seeds were sown into the seedbed, and 200 oilseed rape plants were transplanted into the experimental pots after 30 days. Forty days after transplant, the oilseed rape leaves were suitable for *Sclerotinia sclerotiorum* infection. *Sclerotinia sclerotiorum* was cultured on a potato dextrose agar. The oilseed rape plants were kept in a controlled environment at a temperature of 20 °C and 80% humidity. Two experiments were conducted. For the first experiment, the oilseed rape leaves were inoculated with *Sclerotinia sclerotiorum*. Seventy-two hours later, when the disease symptoms on the leaves became visible, 60 infected leaves and 60 healthy leaves were collected and placed in an icebox to keep the leaves fresh. After the measurement of physiological parameters, the remaining leaves were dried in an oven at a temperature of 75 °C for 48 h. The dried leaves were then ground into a powder, sieved through a 100-mesh sieve, and stored in plastic bags. Seven days later, the second experiment (similar to the first) was conducted.

### Mid-infrared spectra acquisition

The mid-infrared spectra of samples were acquired by a Jasco FT/IR-4100 spectrometer (Japan) in the spectral range of 400–4000 cm^−1^. Before spectra collection, the potassium bromide (KBr) powders were dried in an oven at a temperature of 105 °C for 4 h. Then, 10 mg of each sample was mixed with 490 mg KBr powders, and the mixture was ground and mixed thoroughly. The mixture was then placed into a tablet machine for tabletting, and the sample tablets were used for transmittance mid-infrared spectral data collection. For each sample, 32 scans were applied with a resolution of 8 cm^−1^, and the average of the 32 spectra was used as the transmittance spectrum of the sample.

### Multivariate data analysis

#### Spectra preprocessing

The acquired mid-infrared spectra contained noises. An effective reduction in noises is significant for further analysis. Wavelet transform (WT) is an efficient denoising method in the spectral analysis [12]. WT with mother wavelet Daubechies was applied in this study to reduce the noises.

#### Principal component analysis

Principal component analysis (PCA) is a generally used method for feature extraction and qualitative analysis of the samples. PCA linearly transforms the original data into new orthogonal variables (called principal components, PCs). The first few PCs contains the maximum feature information, which could be used to observe the distribution of samples and identify their differences [13].

#### Classification models

To evaluate the performance of using mid-infrared spectroscopy for identifying the infected and healthy leaves of oilseed rape, we used the partial least square-discriminant analysis (PLS-DA) [14], support vector machine (SVM) [15] and extreme learning machine (ELM) [16] to establish the classification models.

PLS-DA is a widely used supervised pattern recognition method in spectral data analysis. PLS-DA is conducted in the manner of PLS regression (PLSR), with the integral category value as Y variables. PLSR linearly transforms the original data into new variables (called latent variables, LV), and the first few LVs carry the most useful information. The outputs of PLSR and PLS-DA are real numbers with decimals. Thus, the threshold value is needed to determine the category of the samples. Herein, the threshold value was set as 0.5.

SVM is also a widely used supervised pattern recognition method in spectral data analysis. The general concept of SVM is to transform the original data from the low dimension space to the high dimension space, and constructs a hyperplane to maximize the separation of the different sample classes. SVM could address linear and non-linear issues efficiently. The selection of the kernel function is important in SVM. In this study, radial basis function (RBF) was selected as the kernel function.

ELM is a feedforward neural network with a single hidden layer. ELM has shown advantages such as fast learning speed and good generalization ability. In ELM, only the number of neurons in the hidden layer should be set. The determination of the number of neurons in the hidden layer is critical in ELM. In this study, the number of neurons in the hidden layer was determined by a step by step search within a predefined range. The number of neurons corresponding to the best performance was selected.

### Optimal wavenumber selection

The acquired mid-infrared spectra contained a large number of wavenumber variables, which may suffer from the risk of non-informative variables and variable collinearity. With a large number of wavenumber variables, the calibration models may become unstable, computation consuming, complex and difficult to interpret.

Wavenumber (wavelength) selection in spectral analysis for multivariate analysis is an important step in selecting the informative and noncollinear wavenumber variables. The wavenumber (wavelength) selection may improve the model performance while significantly reducing the number of variables, resulting in stable, simple and accurate models.

Second derivative spectrum (2nd spectrum) is a manual selection method based on the spectral profile of the samples [17]. The second derivative is generally used as an efficient preprocessing method in spectral analysis. Compared with the raw spectra, the 2nd spectra could improve spectral resolution, identify overlapping peaks and reduce the background information. Thus, the variables related to the chemical compositions were enhanced and highlighted as peaks and valleys within the 2nd spectra. Therefore, the peaks and valleys with differences between different sample classes were selected as the optimal wavenumbers.

#### Software and model evaluation

In this study, the second derivative preprocessing, PCA and PLS-DA were conducted on the Unscrambler^®^ 10.1 (CAMO AS, Oslo, Norway). The WT preprocessing, SVM and ELM models were conducted on MATLAB (R2014b) software (The Math Works, Inc., Natik, MA, USA). The model performances were evaluated by the classification accuracy in the calibration set and the prediction set.

## Results and discussion

### Mid-infrared spectra

**Fig. 1:**
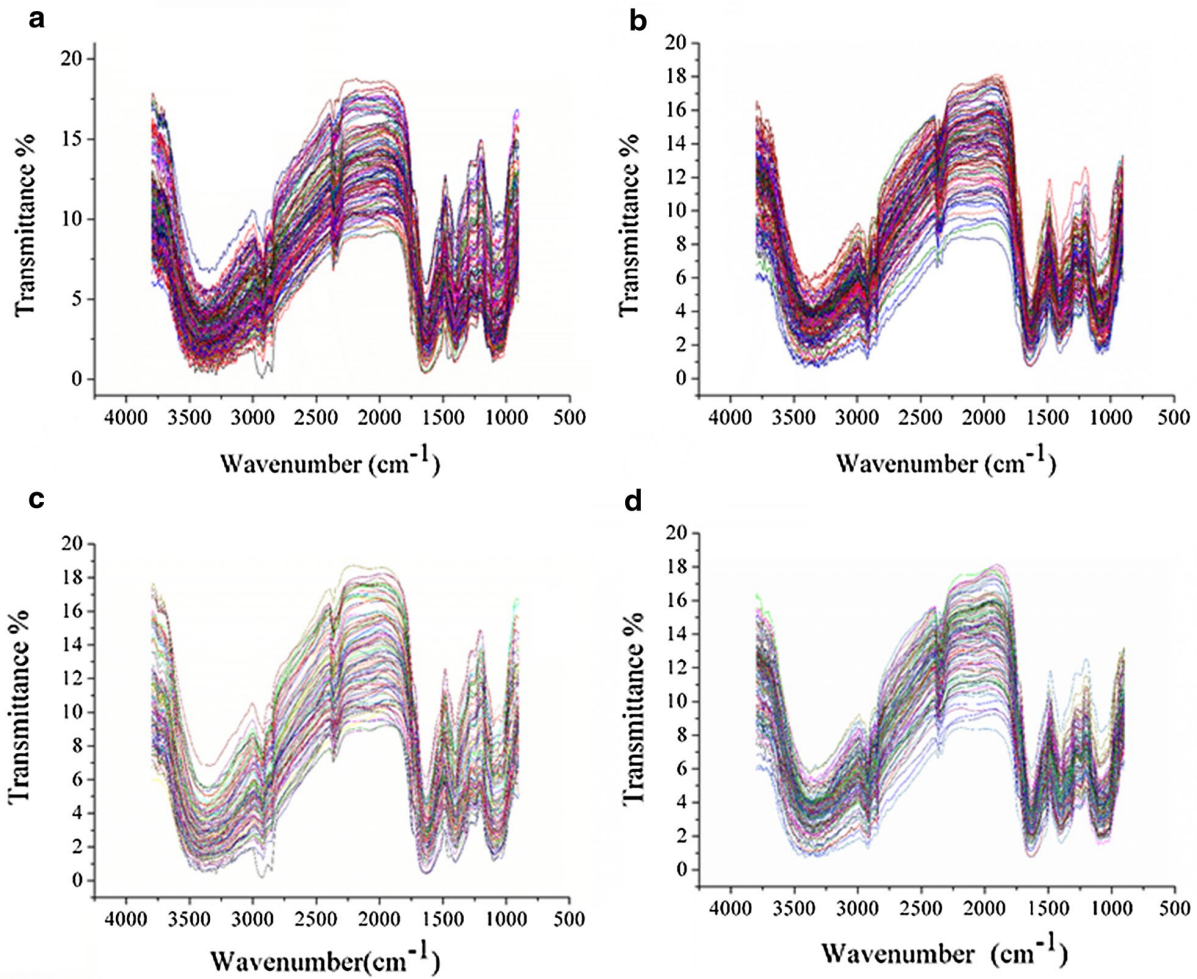
Raw and WT preprocessed spectra of sample set 1 and 2: raw spectra of sample set 1 (**a**), WT preprocessed spectra of sample set 1 (**c**), raw spectra of sample set 2 (**b**), WT preprocessed spectra of sample set 2 (**d**). The differences of raw and preprocessed spectra could be observed

Due to the instrument and experiment conditions, the head and tail of the collected mid-infrared spectra contained obvious noises. Thus, only the spectra in the range of 900–3800 cm^−1^ were studied. Figure 1a, b show the raw spectra of the sample set 1 and 2, and noise could be observed in the two sets. WT was applied on raw spectra to reduce the noise. For sample set 1, WT using Daubechies 6 with a decomposition level of 5 was applied. For sample set 2, WT using Daubechies 5 with a decomposition level of 5 was applied. Figure 1c, d show the preprocessed spectra of the sample set 1 and sample set 2. Obvious denoising could be found in Fig. 1. The general spectral features of sample sets 1 and 2 were similar.

**Fig. 2:**
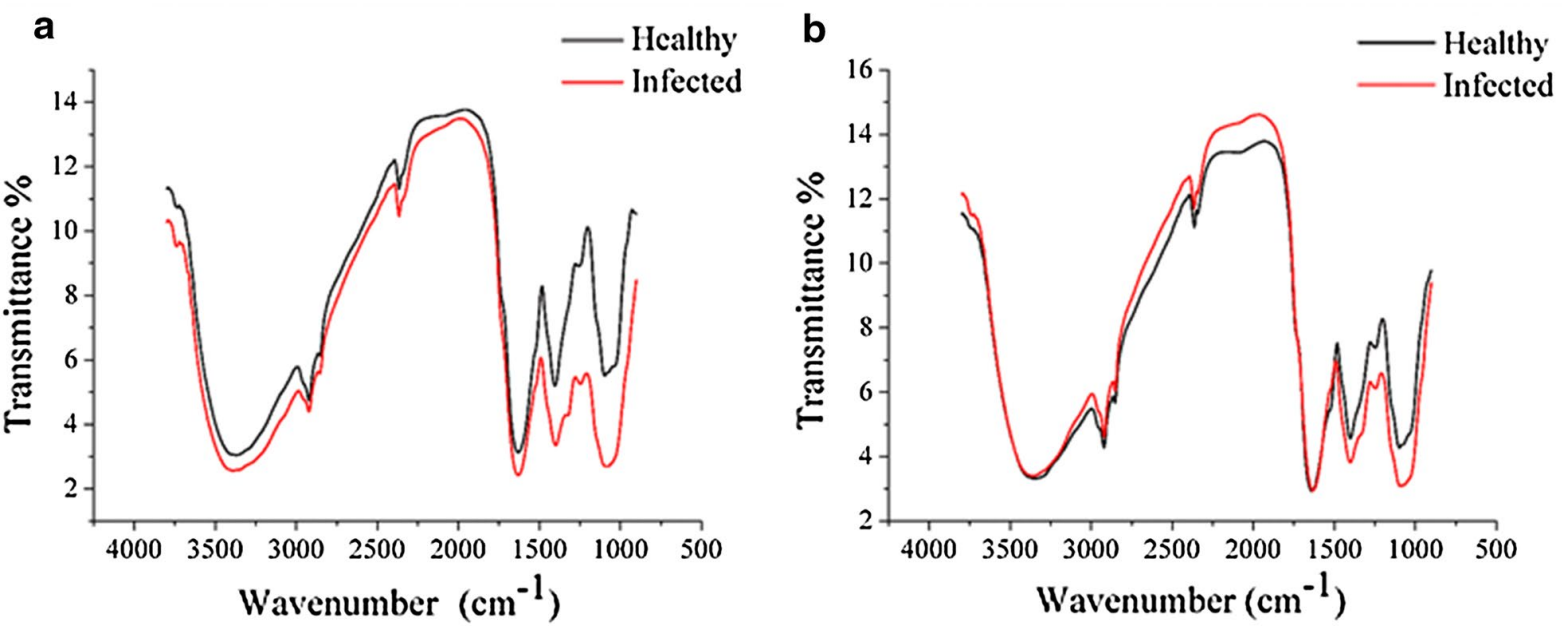
Average spectra of healthy and infected leaves of sample set 1 (**a**), and average spectra of healthy and infected leaves of sample set 2 (**b**). The differences of healthy and infected leaves of two sample sets could be observed

Figure 2a, b show the average spectra of healthy and infected leaves of sample sets 1 and 2. As detailed in Fig. 2a, the average transmittance spectra of healthy and infected leaves of sample set 1 showed differences in their transmittance value, and larger differences could be observed in the ranges of 900–1500 and 1800–2750 cm^−1^. As shown in Fig. 2b, the average transmittance spectra of healthy and infected leaves of sample set 2 showed differences in the transmittance value, and larger differences could be observed in the range of 900–1500 cm^−1^. The larger differences between the healthy and infected leaves of the two types of samples were observed in the ranges of 900–1500 and 1800–2750 cm^−1^, the same as sample set 1.

### PCA analysis

The samples of the sample sets 1 and 2 were randomly divided into the calibration and prediction sets at a ratio of 2:1. The healthy leaves were assigned the category value 1, and the infected leaves were assigned the category value 2.

**Fig. 3:**
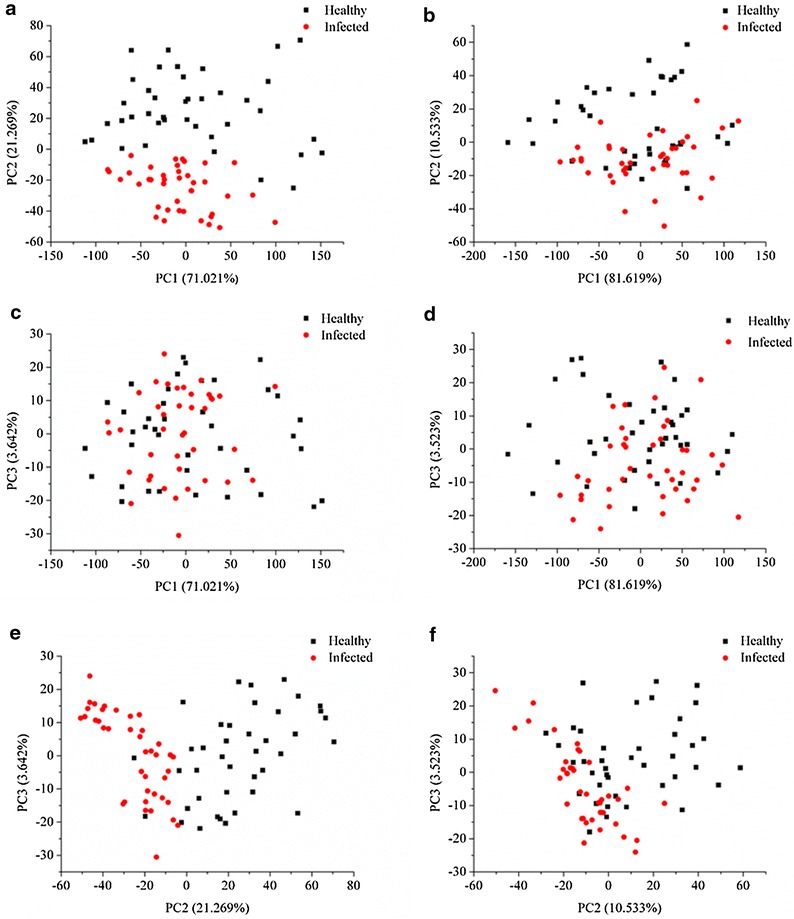
Scores scatter plot of PC1 versus PC2 (**a**), PC1 versus PC3 (**c**) and PC2 versus PC3 (**e**) of sample set 1, and scores *scatter plot* of PC1 versus PC2 (**b**), PC1 versus PC3 (**d**) and PC2 versus PC3 (**f**) of sample set 2. The plots were used to explore the separability between healthy and infected samples qualitatively

PCA was performed on the preprocessed spectra of the calibration set of the sample sets 1 and 2 to visualize the distribution of healthy and infected samples. For sample set 1, PC1, PC2 and PC3 explained 71.021, 21.269 and 3.642% of the total variance, respectively. The first 3 PCs explained 95.931% of the total variance. The score scatter plots of PC1 and PC2, PC1 and PC3, and PC2 and PC3 are shown in Fig. 3a, c, e. Figure 3a, e demonstrated that the healthy samples could be easily differentiated from the infected samples.

For the sample set 2, PC1, PC2 and PC3 explained 81.619, 10.533 and 3.523% of the total variance, and the first 3 PCs explained 95.675% of the total variance. The scores scatter plots of PC1 and PC2, PC1 and PC3, and PC2 and PC3 are shown in Fig. 3b, d, f. Figure 3b, f indicate that the healthy samples could be differentiated from the infected samples with a few overlaps.

PCA of sample set 1 and 2 indicated that healthy and infected leaves could be classified. The distribution of healthy and infected samples in the corresponding score scatter plot of the two sample sets were similar, and due to different sample sets, the separation differed. The distribution of healthy samples in the score scatter plot was observed to disperse more widely than the infected samples.

### Discriminant models using full mid-infrared spectra

PCA provided visual distribution trends of samples, and the discriminant models were further needed for quantitative classification.

PLS-DA, SVM and ELM models were built by using the full mid-infrared spectra of the 2 sample sets to classify the healthy and infected leaves. A PLS-DA model was built using leave-one-out cross validation, and the number of optimal LVs was determined. SVM used RBF as the kernel function, and the optimal penalty coefficient (*C*) and the kernel function parameter gamma (*g*) were obtained by a grid-search procedure in the range of 2^−8^ to 2^8^. The number of neurons in the hidden layer of ELM models were determined by comparing the performances of the ELM models by using different numbers of neurons from 1 to 80 with a step of 1. The ELM models with optimal performances were selected. The results of the discriminant models are shown in Table 1.

**Table 1:** Results of discriminant models using full mid-infrared transmittance spectra of sample sets 1 and 2

Models	Sample set 1			Sample set 2		
	Par^a^	Cal^b^ (%)	Pre^c^ (%)	Par	Cal (%)	Pre (%)
PLS-DA	4	100	85	10	100	100
SVM	(1.7411, 0.0118)	100	80	(84.4485, 0.0039)	100	92.5
ELM	22	100	92.5	60	92.5	90

^a^Par means the parameters of the models, the number of LVs for PLS-DA, (C, g) for SVM and number of neurons for ELM

^b^Cal means the calibration set

^c^Pre means the prediction set

For the sample set 1, all discriminant models demonstrated good performances, with classification accuracies of 100% in the calibration set and over 80% in the prediction set. ELM showed the best results, with a classification accuracy of 92.5%. For the sample set 2, all discriminant models demonstrated good performances, with classification accuracies over 90% in both the calibration and the prediction sets. PLS-DA models showed best results, with classification accuracies of 100% in the calibration and prediction sets.

The performances of the discriminant models in a sample set were different, and the discriminant results of a discriminant model between the 2 sample sets were also different. All discriminant models showed good performances, the sample sets affected the classification performances, and the selection of suitable discriminant models for practical application was imperative.

The discriminant results of the calibration sets of the two sample sets matched with the PCA analysis, and the general discriminant results of the calibration set of the sample set 1 performed slightly better than the calibration set of the sample set 2. Contrarily, the general prediction results of the sample set 2 were slightly better than those of sample set 1. The results were obtained due to the random division of the samples into the calibration and the prediction sets. The overall results indicated that it was feasible to detect SSR on oilseed rape leaves by using mid-infrared spectroscopy, and its practical application in detecting plant diseases was promising.

### Optimal wavenumber selection

In this study, the mid-infrared spectra were acquired with a spectral resolution of 8 cm^−1^. In the spectral range of 900–3800 cm^−1^, there were 1504 wavenumber variables of the spectra. The selection of the informative wavenumber variables was important for better models.

**Fig. 4:**
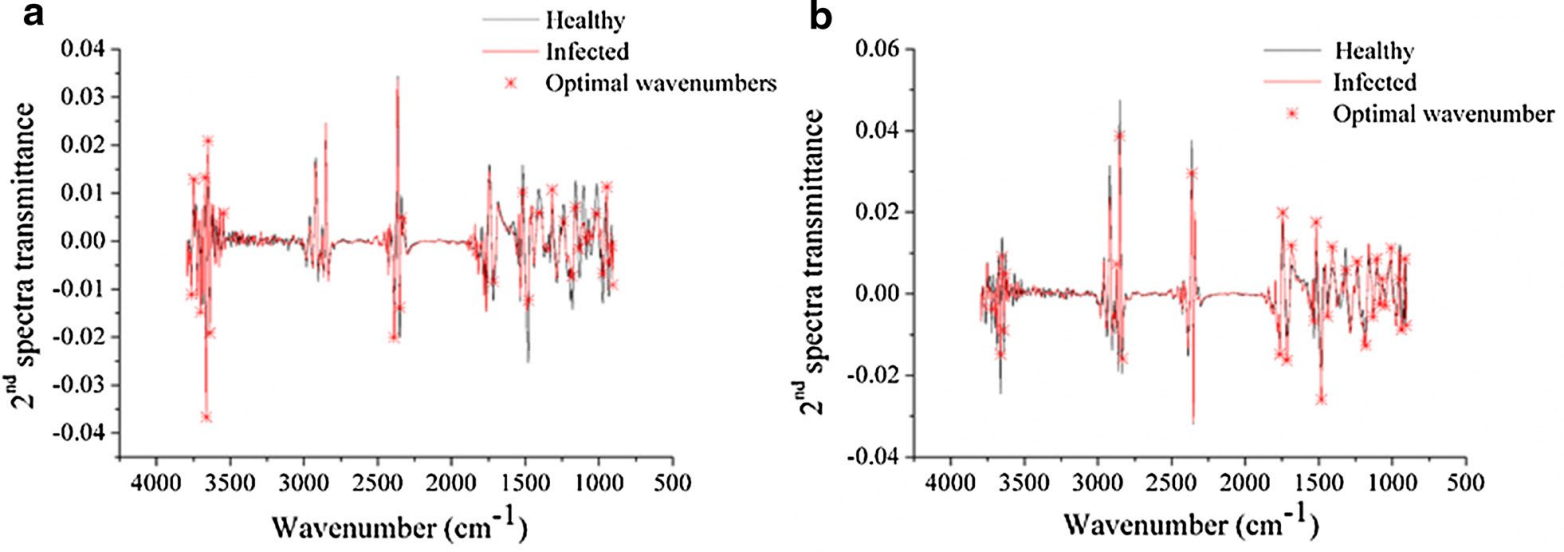
Optimal wavenumbers selected by 2nd spectra of sample set 1 (**a**), and optimal wavenumbers selected by 2nd spectra of sample set 2 (**b**). The *marked peaks* were corresponded to peaks with greater differences, which could be selected and used to discriminant

The second derivative with 7 smoothing points by the Savitzky–Golay algorithm was applied to the average spectra of the healthy and infected leaves of sample sets 1 and 2. The 2nd spectra were used to select optimal wavenumbers. Figure 4 show the 2nd spectra and the corresponding selected optimal wavenumbers of sample sets 1 and 2. The selected wavenumbers are also shown in Table 2.

**Table 2:** Optimal wavenumbers selected by the 2nd spectra of sample sets 1 and 2

	Number	Wavenumber (cm^−1^)
Sample set 1	28	906.3795, 916.0218, 935.3065, 946.8773, 973.8758, 1018.2305, 1070.2992, 1083.7985, 1133.9386, 1159.0087, 1180.2218, 240.0043, 1317.1429, 1409.7094, 1479.1342, 1517.7035, 1716.3356, 2341.1589, 2350.8013, 2389.3706, 3546.4507, 3639.0171, 3650.5879, 3662.1587, 3671.801, 3700.728, 3747.0112, 3762.439
Sample set 2	31	906.3795, 916.0218, 937.2349, 948.8057, 1008.5882, 1049.0861, 1070.2992, 1085.7269,1105.0116, 1132.0101, 1180.2218, 240.0043, 1317.1429, 1409.7094, 1440.5648, 1481.0626, 1517.7035, 1533.1312, 1685.4801, 1716.3356, 745.2626, 1764.5472, 2362.3721, 2834.8464, 2852.2026, 2875.3442, 3629.3748, 3639.0171, 3652.5164, 3662.1587, 3721.9412

Figure 4 and Table 3 demonstrate that the 2nd spectra of the sample sets 1 and 2 were quite similar, and the maximum optimal wavenumbers selected by the 2nd spectra of the 2 sample sets were similar or the same. Some differences were also observed due to the variations among the different sample sets and the instrument condition. The selected optimal wavenumbers matched the spectra regions with differences of the average spectra shown in Fig. 2.

**Table 3:** The results of the discriminant models using optimal wavenumbers from sample sets 1 and 2

Models	Sample set 1			Sample set 2		
	Par	Cal (%)	Pre (%)	Par	Cal (%)	Pre (%)
PLS-DA	6	100	82.5	9	100	100
SVM	(5.2780, 1)	100	82.5	(48.5029, 0.0359)	95	95
ELM	62	100	95	76	100	95

The optimal wavenumbers selected by the 2nd spectra of the 2 sample sets showed repeatability, indicating the efficiency for the optimal wavenumber selection by 2nd spectra. However, the number of samples used for optimal wavenumber selection were small, which was common in spectral analysis. More samples were needed to obtain the optimal wavenumbers for practical applications. The selection of optimal wavenumbers in the 2 sample sets indicated the possibility of selecting the widely accepted optimal wavenumbers by the 2nd spectra for practical application.

The selected peaks in the 900–1200 cm^−1^ region were attributed to the C–O stretching bands mainly from the carbohydrates [18], whereas those in the 1500–1700 cm^−1^ region were attributed to the amide bands of proteins [18]. The selected peaks in the 1200–1500 cm^−1^ region were assigned to the C–H bending modes [19]; the peak at 1716.336 cm^−1^ was assigned to the amide I of protein [20]; the peak at 1745.263 cm^−1^ was assigned to the COOR bond [17]; the peak at 1764.547 cm^−1^ was attributed to the symmetric C=O stretching of the ester group [21]; the peak at 2350 cm^−1^ was assigned to the asymmetric C=O bonds [22]. Moreover, peaks in the 2800–3000 cm^−1^ region were attributed to the lipid [23], and those in the 3000–3800 cm^−1^ region were attributed to the O–H stretching vibrations [24].

### Discriminant models using optimal wavenumbers

To evaluate the performance of the selected optimal wavelengths in SSR detection, PLS-DA, SVM and ELM models were built. The modelling procedure was the same with the full spectra models. The results of the discriminant models are illustrated in Table 3.

For the sample set 1, all discriminant models showed good performances, with classification accuracies of 100% in the calibration set and over 80% in the prediction set. ELM models performed the best with a classification accuracy of 95% in the prediction set.

For the sample set 2, all discriminant models demonstrated satisfactory performances, with classification accuracies over 95% in both the calibration and the prediction sets. PLS-DA models performed best with a classification accuracy of 100% in both the calibration and the prediction sets.

Notably, the performances of the discriminant models using the optimal wavenumbers were different. Nevertheless, all discriminant models showed good performances.

The results of optimal wavenumber selection and the calibration models using the selected optimal wavenumbers of the 2 sample sets suggested the efficiency and the reliability of the optimal wavenumber selection, indicating a great potential for practical application.

### Comparison of the full spectra models and optimal wavenumber models

As presented in Tables 1 and 3, the discriminant models using the full spectra and the selected optimal wavenumbers all showed good performances. For the sample set 1, the discriminant models using the optimal wavenumbers showed similar results as the discriminant models using full spectra. However, the number of wavenumber variables was reduced from 1504 to 28, resulting in a reduction of 98.138%. These results indicated that optimal wavenumbers selected by the 2nd spectra could significantly reduce the number of wavenumber variables in the mid-infrared spectra, and the selected optimal wavenumbers were capable of keeping the model performances for sample set 1. For sample set 2, the discriminant models using the optimal wavenumbers showed similar results as the discriminant models using full spectra. Nonetheless, the wavenumber variables were reduced from 1504 to 31, resulting in a reduction of 99.939%. The results indicated that the optimal wavenumbers selected by the 2nd spectra could significantly reduce the number of wavenumber variables in the mid-infrared spectra, and the selected optimal wavenumbers were capable of keeping the model performances for sample set 2.

Considering that the optimal wavenumbers selected by the 2nd spectra for the 2 sample sets were similar, and the models using the optimal wavenumbers of the 2 sample sets showed good performances, mid-infrared spectroscopy combined with optimal wavenumber selection by the 2nd spectra was proven to be an efficient and promising technique for SSR detection of oilseed rapes. However, beyond the exploration and validation of using the mid-infrared spectroscopy combined with chemometrics for detecting plant diseases, the results of this study also indicated that mid-infrared spectroscopy was an efficient, reliable and promising technique with practical applications, and not just the feasibility of exploration.

## Conclusion

Two sample sets of SSR infected oilseed rape leaves and their corresponding mid-infrared transmittance spectral information were studied to detect SSR on oilseed rape leaves. The differences in the mid-infrared spectra of the healthy and infected leaves indicated the differences in their physiological constituents in the corresponding samples. The discriminant results by different models indicated the feasibility of using mid-infrared spectra for detecting SSR on oilseed rape leaves. The results of discriminant models (including PLS-DA, SVM and ELM) and the optimal wavenumber selection method (2nd spectra), showed the effectiveness of mid-infrared spectroscopy combined with chemometrics in detecting SSR on oilseed rape leaves. The quite similar optimal wavenumbers selected by the 2nd spectra demonstrated the effectiveness of wavenumbers selection. The results of the 2 sample sets proved and validated that mid-infrared spectroscopy was a promising and reliable technique for SSR detection. Mid-infrared spectroscopy could be an efficient method for disease detection for real-world disease control, with a reliable and accurate selection of optimal calibration models and optimal wavenumbers.
